# Aroma evolution of Anhua Qianliang tea across different storage years: Machine learning-assisted discrimination of storage stages and screening of marker volatile compounds

**DOI:** 10.1016/j.fochx.2026.104151

**Published:** 2026-06-29

**Authors:** Mengzhen Xia, Zhichao Lin, Guohe Chen, Jiazhen San, Xiaoxiao Fan, Lianqing Wang, Jianan Huang, Zhonghua Liu, Chao Wang

**Affiliations:** a Key Laboratory of Tea Science of Ministry of Education, Hunan Agricultural University, Changsha 410128, China; b National Research Center of Engineering and Technology for Utilization of Botanical Functional Ingredients, Changsha 410128, China; c State Key Laboratory of Tea Plant Germplasm Innovation and Resource Utilization, Hunan Agricultural University, Changsha 410128, China; d Key Laboratory for Evaluation and Utilization of Gene Resources of Horticultural Crops, Ministry of Agriculture and Rural Affairs of China, Hunan Agricultural University, Changsha 410128, China; e Tea Cultivar Innovation Center, Yuelushan Laboratory, Changsha 410128, China

**Keywords:** Anhua Qianliang tea, Multivariate statistics, Machine learning, Sensory-volatile correlation

## Abstract

Anhua Qianliang tea (QLT) is a representative compressed dark tea whose aroma quality is progressively remodeled during long-term storage, yet the volatile basis underlying this transition remains poorly understood. In this study, quantitative descriptive analysis (QDA), GC × GC-QTOFMS, multivariate statistics, and machine learning were integrated to resolve the sensory evolution, volatile remodeling, and stage-related aroma markers of QLT across different storage years. Aging shifted the aroma profile of QLT from green and fresh notes in the early stage to woody, stale, and herbal characteristics in the late stage. Volatile profiling identified 281 compounds, and storage-stage differentiation was mainly associated with coordinated changes in lipid oxidation-derived volatiles, carotenoid-derived compounds, oxygenated terpenoids, and methoxybenzene derivatives. Correlation analysis further linked lipid-derived volatiles to early-stage aroma expression, whereas carotenoid-derived compounds, oxygenated terpenoids, and methoxybenzene derivatives were more closely associated with aged aroma. Differential screening identified linalool, *β*-ionone, dihydroactinidiolide, safranal and other compounds as potential stage-related markers. Machine learning further showed that CatBoost achieved the best classification performance, while SHAP analysis highlighted octanal, hexanal, (*Z*)-4-heptenal, among others, as major contributors to storage-stage discrimination. Together, these results support a stage-dependent volatile remodeling framework for QLT aging and provide a chemical basis for storage-stage identification and aroma quality evaluation.

## Highlights

• Aging shifted QLT aroma from green and fresh to woody, stale, and herbal.
• Correlation analysis resolved key odorants linked to aging.
• Twelve key differential volatiles discriminated early and late stages.
• CatBoost showed the best performance for storage-stage classification.
• Machine learning improved stage discrimination and marker discovery.

## Graphical abstract

**Figure:**
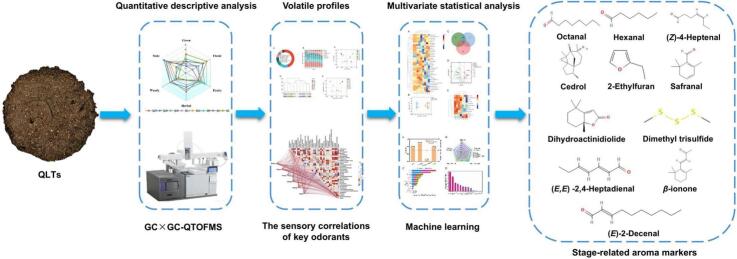

## 1 Introduction

Tea is one of the most widely consumed beverages worldwide. Dark tea, a distinctive category of tea, derives its name from the unique pile-fermentation and drying process that yields its characteristic dark and lustrous appearance (Wang, Huang, et al., 2025). Major dark tea products in China include Anhua dark tea from Hunan, Pu-erh tea from Yunnan, Tibetan tea from Sichuan, Liubao tea from Guangxi, and Qingzhuan tea from Hubei. Anhua Qianliang tea (QLT) is a traditional representative product of Anhua dark tea. It is named after the traditional net weight of each tea roll, which is 1000 liang in the old Chinese weight system. It is also known as “Huajuan tea” because the outer bamboo basket is woven into a lattice pattern. It is produced from dark tea raw materials through a series of processes, including screening, steaming, basket filling, pressing, shaping, and natural drying. A distinctive feature of QLT is that tea processing and packaging are completed simultaneously. During pressing and shaping, the tea leaves are packed into long cylindrical bamboo baskets lined with Polygonum leaves and brown leaves. The baskets are then wrapped with bamboo strips and repeatedly pressed, pounded, and twisted, ultimately forming a compact cylindrical tea body. Compared with conventional compressed teas, this multilayer packaging, composed of natural plant materials, is not only an integral part of the final structure but also creates a unique microenvironment for slow drying and long-term aging. This packaging system is therefore considered a key factor in the development of the quality characteristics and flavor of QLT (Huang et al., 2024). Specifically, the multi-layer packaging constructed by polygonum leaves, brown leaves, and bamboo baskets creates a unique semi-closed micro-environment that subtly regulates moisture retention and oxygen permeability. This micro-environment selectively facilitates the growth and succession of functional microbial communities (such as Eurotium and Aspergillus species) during the aging process (Huang et al., 2024; Li et al., 2018). These microorganisms secrete extracellular enzymes to catalyze the degradation of polyphenols and macromolecules. Furthermore, the slow interaction between the tea matrix and these plant-based packaging materials under this semi-anaerobic condition promotes complex non-enzymatic oxidations and potentially provides additional precursors for volatile compound accumulation, driving the formation of the characteristic woody and herbal aromas in QLT (Shi et al., 2021). Owing to its distinctive appearance, elaborate manufacturing process, long history, and high quality, QLT has long been one of the most popular and best-selling products of Hunan dark tea.

Aged tea has attracted considerable attention because of its unique flavor characteristics, which distinguish it from freshly produced tea. Among the sensory attributes of tea, aroma is a key component in the comprehensive evaluation of tea quality and flavor (Wang, Zhang, et al., 2025). Each type of dark tea exhibits distinctive aroma characteristics. For example, Tibetan tea is characterized by oily notes, Pu-erh tea typically presents an aged aroma, and Liupao tea is known for its aged, woody, and betel nut-like notes. In addition, prolonged storage can lead to significant changes in the aroma profile of tea leaves (Liu, Shen, et al., 2025). During long-term storage, the aroma profile of Tuo tea gradually shifts from fresh, fruity, and floral notes toward more aged, woody, and herbal characteristics. Moreover, theaspirane, eucalyptol, *o*-xylene, and 1-ethylidene-1H-indene have been identified as potential chemical markers associated with Tuo tea aging (Chen, Liu, et al., 2024). During prolonged storage, the aroma profile of raw Pu-erh tea gradually shifts from fruity and floral characteristics to smoky and aged attributes (Liu, Wang, et al., 2025). After storage, the aroma profile of Bai Mudan tea gradually shifts from fresh characteristics, represented by green and fruity notes, to aged characteristics, such as woody and stale notes. Cedrol and isophorone have been identified as characteristic aroma compounds contributing to the aged aroma of Bai Mudan tea (Li et al., 2026). By integrating GC-*E*-NOSE, GC–MS, and GC-IMS analyses, nine volatile compounds were identified as key markers for discriminating mature Pu-erh teas with different storage ages (Fang et al., 2024). Current evidence indicates that tea aging is influenced by multiple factors, including storage duration, temperature, humidity, and packaging conditions (Wang et al., 2025). Research on the aroma changes of QLT during storage is relatively scarce, and due to its unique packaging system and manufacturing process, its aging behavior is particularly complex. Therefore, a systematic investigation of the quality changes of QLT during storage is of considerable importance.

While traditional linear methods like Partial Least Squares Discriminant Analysis (PLS-DA) are useful for preliminary pattern recognition, they often struggle to capture the highly complex, non-linear metabolic fluctuations inherent in tea aging, and can be susceptible to overfitting with high-dimensional volatilomics data (Gromski et al., 2015). In recent years, machine learning as a new potent tool for food VOC data analysis, offering efficient, accurate, fast prediction and classification capabilities to overcome current challenges in food industry (Feng et al., 2024). Explainable artificial intelligence (XAI) tools such as Shapley Additive Explanations (SHAP) improve model transparency by linking predictions to underlying features (Ge et al., 2025). ML models further enhance both prediction accuracy and generalizability. In the tea field, machine learning and computer vision have been increasingly applied to objective quality assessment, grade discrimination, and multidimensional sensory evaluation, showing considerable potential for improving consistency, efficiency, and interpretability in tea research and industry (Wu et al., 2025; Chen, Zhu, et al., 2024). Therefore, combining machine learning with volatilomics and sensory analysis may provide a useful complementary approach for storage-stage discrimination and marker volatile compound screening in QLT.

Therefore, this study employed quantitative descriptive analysis (QDA) combined with GC × GC-QTOFMS to investigate the sensory changes and volatile evolution of QLT across different storage years. Sensory-volatile correlation analysis was further conducted to explore the associations between aroma attributes and key volatile compounds, while multivariate statistical analysis and machine learning were applied to discriminate storage stages and screen marker volatile compounds associated with storage-year variation. This study aimed to provide a theoretical basis for optimizing storage conditions and understanding aging-related quality formation in QLT.

## 2 Materials and methods

### 2.1 Tea samples and chemical reagents

Anhua Qianliang tea (QLT) samples from nine production years (2000, 2005, 2007, 2011, 2013, 2016, 2019, 2021, and 2023) were obtained from Hunan Liyuanlong Tea Industry Co., Ltd. (Anhua, Hunan, China). At the time of analysis, the corresponding storage durations were 25, 20, 18, 14, 12, 9, 6, 4, and 2 years, respectively. For convenience, the samples were coded according to storage duration using the prefix “Q”; thus, the sample stored for 25 years was designated as Q25. All samples were produced according to the manufacturer's standardized protocols using the same raw materials and processing techniques, including screening, steaming, basket packing, pressing, shaping, and natural drying. After production, the samples were stored under natural environmental conditions in the manufacturer's warehouse in Anhua, Hunan, China, where the average annual temperature was 16.6 °C and the average annual relative humidity was 80.7%. Due to the extreme rarity and high cost of authentic aged QLT across 9 storage years, obtaining multiple independent whole tea columns per year was practically unfeasible. Therefore, this study relied on technical replicates from one highly representative tea column carefully selected for each year. While this approach effectively captures the specific evolutionary trends of these representative samples, future validation with larger biological cohorts is necessary.

N-alkanes (C_7_-C_28_) were purchased from Sigma-Aldrich (Shanghai, China).

### 2.2 Quantitative descriptive analysis of the aroma

Sensory evaluation was conducted by eight well-trained panelists from Hunan Agricultural University (four males and four females, aged 23–61 years) in accordance with the Chinese national standard, “Methodology of Sensory Evaluation of Tea” (GB/T 23776–2018). The aroma profile was characterized using quantitative descriptive analysis (QDA) (Chen et al., 2024). Briefly, 3 g of each tea sample was infused with 150 mL of boiling water for 8 min in a dedicated tea evaluation cup, and the infusion was then decanted. The panelists evaluated the aroma of the infused leaves remaining in the cup. The aroma attributes were established by consensus among the eight panelists based on the sensory characteristics of QLT and included green, floral, fruity, stale, woody, and herbal notes. Prior to formal scoring, the panelists underwent systematic training sessions. To ensure objective evaluation, widely accepted sensory reference materials corresponding to these six attributes were utilized as standardized anchors to define and calibrate the 5-point intensity scale (where 0 indicated no perception, 3 indicated medium intensity, and 5 indicated very strong intensity). Through repeated group discussions and calibration tests using these reference anchors, the panel aligned their sensory perceptions and established a strong consensus on the scoring criteria. All evaluations were performed in a quiet, odor-free environment. Each sample was evaluated three times on different days, and the final score for each attribute was expressed as the mean of the three replicates. Prior to the sensory testing sessions, all panel members received comprehensive information regarding the research aims, the evaluation protocol, and the planned application of the data. The ethics committee of Hunan Agricultural University waived the requirement for formal ethical approval because the study solely involved the routine tasting of commercially available and safe tea products devoid of any medical or physiological interventions. Written informed consent was secured from every evaluator beforehand. Engagement in this sensory panel was strictly voluntary. To guarantee confidentiality, all sensory findings were completely anonymized and are reported exclusively as aggregate data. No personal identifiers of the participants are revealed in the main text or the associated supplementary materials.

### 2.3 Volatile compounds detected by HS-SPME-GC × GC-QTOFMS

The HS-SPME procedure was carried out according to an optimized method previously established by our research team (Ouyang et al., 2025). Briefly, 0.500 g of powdered tea sample was accurately weighed into a 20 mL headspace vial. The vial was placed in the autosampler tray of a CTC system, which automatically added 10 μL of ethyl decanoate (8.64 mg/L) as the internal standard and 5 mL of boiling water. The sample was then equilibrated at 60 °C with agitation at 600 rpm for 10 min. After equilibration, a 50/30 μm DVB/CAR/PDMS fiber was exposed to the headspace for 30 min under the same temperature and agitation conditions. The fiber was finally thermally desorbed in the GC inlet at 250 °C for 10 min.

Volatile compounds were analyzed using comprehensive two-dimensional gas chromatography coupled with quadrupole time-of-flight mass spectrometry (GC × GC-QTOFMS; Agilent Technologies, Santa Clara, CA, USA) equipped with an olfactory detection port. The system was fitted with an HP-5MS column in the first dimension and a DB-17MS column in the second dimension, both housed in a single oven and obtained from Agilent Technologies (Santa Clara, CA, USA). The oven temperature program was identical for both dimensions: 40 °C for 1 min, increased at 4 °C/min to 180 °C, and then increased at 20 °C/min to 250 °C and held for 1 min. A solid-state modulator (SSM 1800) was used with a modulation period of 4 s. The mass spectrometer was operated in electron ionization (EI) mode at 70 eV, with the ion source and quadrupole temperatures set at 200 °C and 150 °C, respectively. Mass spectra were acquired over an *m*/*z* range of 45–500 amu. The retention index (RI) was calculated using an n-alkane series. Compounds were retained according to previously established criteria, including a forward match factor > 700, a reverse match factor > 800, and an RI deviation <30 (Chen et al., 2024).

### 2.4 Identification and relative quantification of volatile compounds

The GC × GC-QTOFMS data were processed using Canvas software (version 1.0.0.25117). Volatile compounds were identified by comparing their mass spectra with those in the NIST 20 database. Retention indices (RIs) were calibrated using a C7-C28 n-alkane series. Compound identification was confirmed when all of the following criteria were met: a forward match factor > 700, a reverse match factor > 800, and an RI deviation <30. The statistical comparison tools integrated within the Canvas platform were used to align and compare peak tables across samples. The relative concentrations of volatile compounds were quantified using the internal standard method according to the following formula:

$$C_{i}\;\left(\mu g/\mathrm{kg}\right)=\frac{\mathrm{Ratio}\times 10\;\mathrm{\mu L}\times 8.64\;\mathrm{mg}/L}{M}$$

Note: *Ratio* is the ratio of the peak area of the compound to the peak area of the internal standard; *Ci* is the concentration of each odor compound (μg/kg); *M* is the mass of the tea sample, 0.5 g. Each sample is tested three times.

The contribution of volatile compounds to the overall aroma was evaluated using the relative odor activity value (ROAV). ROAV was calculated as the ratio of the concentration of each compound to its odor threshold in water (Zheng et al., 2025). Compounds with an ROAV greater than 1 were considered potential key aroma contributors. The formula for ROAV is as follows:

$$\text{ROAV}=\frac{\mathrm{Ci}}{\mathrm{Ti}}$$

Note: *Ci* (μg/kg) is the relative content of odor compounds, *Ti* (μg/kg) is the threshold for odor compounds.

### 2.5 Machine learning-based data preprocessing, model selection, and model interpretation

Based on the results of multivariate statistical analysis, the class labels of QLT samples were reassigned according to storage stage. Samples stored for≤10 years were classified as the early stage, those stored for 12 and 14 years as the middle stage, and those stored for 18–25 years as the late stage. Subsequently, *Z*-score normalization was applied to reduce scale-related effects and improve model convergence. The Z-scores for each variable were calculated as follows:

$$X_{i}^{*}=\frac{X_{i}-\mu }{\sigma }$$

where *X*_*i*_^∗^ is the standardized value after normalization, *X*_*i*_ is the raw value of the feature, *μ* is the mean of the feature, and *σ* is the standard deviation of the feature.

The discriminatory ability of volatile compounds across different storage stages was evaluated using four machine learning models: Dummy Classifier, K-nearest neighbors (KNN), support vector machine (SVM), and Categorical Boosting (CatBoost). These models have demonstrated strong performance in food-related classification tasks and were therefore selected for this study (Ge et al., 2025). Model development was carried out using Python-based libraries, primarily scikit-learn, while Matplotlib was used for visualization. Given the relatively limited number of samples in this study, Leave-One-Out Cross-Validation was rigorously employed to evaluate the classification performance of the machine learning models. This stringent validation strategy maximizes data utilization by iteratively using one single sample for testing while training the model on all remaining samples. This approach effectively mitigates the risk of overfitting and provides a highly reliable and unbiased assessment of model accuracy. All evaluation metrics were calculated based on the comprehensive results of this strict cross validation process.

Classification performance was evaluated using Accuracy, Balanced Accuracy, Macro Precision, Macro Recall, and Macro F1-score. Accuracy was defined as the proportion of correctly classified samples among all samples. Balanced Accuracy was calculated as the average recall across classes. For each class, Precision was calculated as TP/(TP + FP), Recall as TP/(TP + FN), and F1-score as 2 × Precision × Recall/(Precision + Recall), where TP, FP, and FN denote true positives, false positives, and false negatives, respectively. Macro Precision, Macro Recall, and Macro F1-score were calculated as the unweighted mean values of the corresponding class-wise metrics.

Shapley Additive Explanations (SHAP) was used to interpret the best-performing machine learning model in this study (Feng et al., 2024). The method was implemented in Python to quantify feature contributions and to examine their relationships with model predictions. SHAP analysis was further performed to evaluate the influence of individual features on prediction outcomes. The SHAP value for feature i was calculated as follows:

$$\phi_{i}=\sum_{S\subseteq F|\left\{i\right\}}\frac{|S|!\left(|F|-|S|-1\right)!}{|F|!}\left[f\left(S\cup \left\{i\right\}\right)-f\left(S\right)\right]$$

where *F* is the set of all features, *S* is a subset of features excluding feature *i*, *f*(*S*) is the model output based on feature subset *S*, and *ϕ*_*i*_ represents the SHAP value of feature *i*. A higher absolute SHAP value indicates a greater contribution of the corresponding volatile compound to the model prediction. Mean absolute SHAP values were used to rank the importance of volatile compounds and to identify marker volatile compounds associated with storage-stage discrimination.

### 2.6 Statistical analysis

Due to the rarity of the historical samples, all instrumental and sensory experiments were performed using three technical replicates from the representative tea column of each storage year to ensure maximum analytical precision. Differential metabolites were screened using the following criteria: variable importance in projection (VIP) score ≥ 1, *p* < 0.05, and |log_2_FC| >1. VIP values were obtained from orthogonal partial least squares-discriminant analysis (OPLS-DA) and partial least squares-discriminant analysis (PLS-DA), while the corresponding score plots and permutation tests were generated using the R package MetaboAnalystR. Heatmap analysis and principal component analysis (PCA) were also performed using MetaboAnalystR. The QDA radar chart was generated using Origin 2024b software (OriginLab Corporation, Northampton, MA, USA).

## 3 Results and discussion

### 3.1 Sensory differences among QLT samples with different storage years

**Fig. 1:**
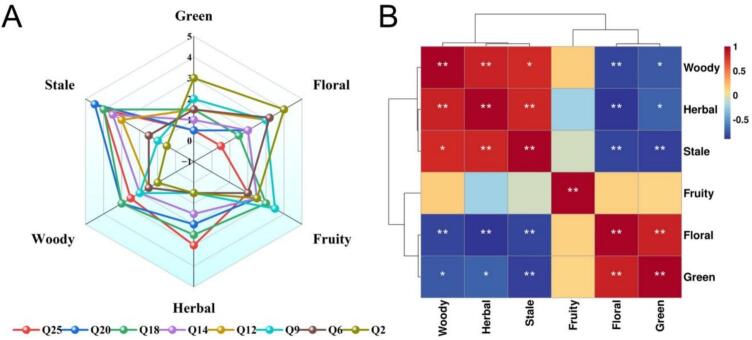
Radar chart and correlation heatmap of aroma attributes in QLT. (A) Radar chart showing the sensory intensity distribution of six aroma descriptors in QLT at different aging stages. (B) Correlation heatmap of sensory aroma attributes in QLT. Red and blue indicate positive and negative correlations, respectively. Asterisks indicate significant correlations (* *p* < 0.05, ** *p* < 0.01). (For interpretation of the references to color in this figure legend, the reader is referred to the web version of this article.)

To evaluate the aroma differences among QLT samples with different storage periods, their sensory characteristics were first assessed by QDA. Six aroma attributes were selected to characterize QLT, including green, floral, fruity, stale, woody, and herbal notes. As shown in Fig. 1A, the aroma profile of QLT changed significantly with prolonged storage, indicating that aging had a marked influence on its sensory quality. In general, younger samples showed higher intensities of green and floral notes, whereas older samples were characterized by more pronounced woody, stale, and herbal attributes (Chen et al., 2024). To further elucidate the dynamics of this stage-dependent variation, a correlation analysis among the sensory attributes was conducted (Fig. 1B). The heatmap revealed two distinct and opposing sensory clusters. The fresh attributes (green and floral) exhibited a strong positive correlation with each other. Conversely, they were significantly negatively correlated with the aged attributes (woody, herbal, and stale), which formed a separate highly positively correlated cluster. This strong antagonistic relationship mathematically substantiates the temporal evolution observed across the storage stages. Specifically, the fresh notes strictly dominate the early storage period. As storage time increases, these initial aromas are progressively replaced by their negatively correlated counterparts. Ultimately, in the late-stage samples (Q18, Q20, and Q25), the fresh notes almost dissipate, while the intensities of the ‘woody’ and ‘stale’ attributes reach their absolute peaks. This explicit association between the sensory correlation clusters and the specific storage periods clearly demonstrates that the long-term aging of QLT is a continuous transition from a fresh profile to profound woody and aged aromas.

### 3.2 Characterization of volatile profiles in QLT across storage years

**Fig. 2:**
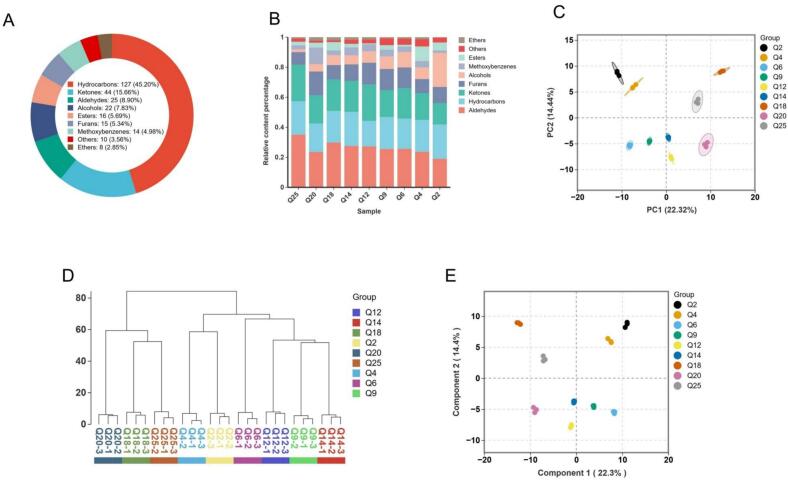
Overall characterization and multivariate analysis of volatile compounds in QLT during aging. (A) Donut chart showing the distribution of identified volatile compounds by chemical class in QLT. (B) Stacked bar chart showing the relative content percentages of different classes of volatile compounds in QLT samples at different aging stages. (C) PCA score plot based on volatile compound profiles of QLT samples at different aging stages. (D) HCA of QLT samples based on volatile compound composition. (E) Partial least squares-discriminant analysis (PLS-DA) score plot showing the discrimination of QLT samples at different aging stages based on volatile profiles.

The volatile compounds of QLT samples stored for different years were characterized by GC × GC-QTOFMS. In total, 281 volatile compounds were identified across all samples. Among these compounds, hydrocarbons were the most abundant class, accounting for 45.20% of the total identified volatiles, followed by ketones (15.66%), aldehydes (8.90%), alcohols (7.83%), esters (5.69%), furans (5.34%), and methoxybenzenes (4.98%) (Fig. 2A). The broad distribution of these compound classes highlights the chemical complexity of the QLT aroma profile. This result is consistent with previous reports indicating that tea aroma is formed by multiple volatile compounds derived from lipids, carotenoids, glycosides, and other precursors. Specifically, lipid-derived aldehydes and furans are generally associated with green, fresh, and fatty notes, whereas carotenoid-derived ketones and related compounds contribute floral, fruity, sweet, and aged aroma characteristics (Ho et al., 2015).

The stacked bar chart further demonstrated that, although all samples contained broadly similar classes of volatile compounds, the relative proportions of these classes varied across storage years (Fig. 2B). Aldehydes and hydrocarbons were the dominant components in all samples. Notably, some younger samples exhibited a relatively higher proportion of aldehydes, whereas methoxybenzene derivatives and certain oxygenated compounds became more pronounced in samples from the middle and late storage stages. These findings suggest that the aging of QLT is not associated with the appearance of entirely new volatile classes, but rather with dynamic changes in the relative abundance and contribution of pre-existing compound groups. Notably, while methoxybenzene derivatives constitute a moderate proportion of the total volatile constituents in QLT, existing literature highlights their potential significance in the aged aroma profile of dark teas (Sheng et al., 2023; Wang et al., 2025). In this study, the gradual increase of these compounds during storage closely follows the development of woody and stale aromas. Although this compositional trend should not be overinterpreted as a direct sensory causation, these derivatives serve as significant chemical signatures of long-term aging in QLT. Consequently, these findings establish a robust chemical foundation for subsequent multivariate discriminant analysis and the identification of reliable markers across distinct storage stages.

To comprehensively evaluate the changes in volatile characteristics among Qianliang tea samples stored for different years, both unsupervised and supervised multivariate analyses were performed using the normalized GC × GC-QTOFMS dataset. Principal component analysis (PCA) and hierarchical cluster analysis (HCA) are commonly used to reveal the overall clustering patterns of tea aroma samples, whereas Partial least squares-discriminant analysis (PLS-DA) is generally employed to improve group discrimination and identify volatile compounds associated with storage- or processing-related differences (Ho et al., 2015; Shen et al., 2023). As shown in the PCA score plot (Fig. 2C), PC1 and PC2 explained 22.32% and 14.44% of the total variance, respectively, with a cumulative contribution of 36.76%. Q2 and Q4 showed a tendency to cluster together, whereas Q6, Q9, Q12, and Q14 formed a relatively close group. In contrast, Q18, Q20, and Q25 were mainly distributed on the right side of the plot. The first two principal components accounted for approximately 36.76% of the total variance. This relatively moderate explained variance suggests that unsupervised linear models are limited in fully capturing the complex data structures within the samples. Such a phenomenon is frequently observed in the long-term aging of dark teas because the dynamic accumulation of volatile compounds involves complex high-dimensional and non-linear metabolic fluctuations. Consequently, linear dimensionality reduction alone cannot entirely resolve these subtle stage-dependent variations. To overcome this limitation and deeply mine the dataset, it is completely necessary to employ further advanced analytical strategies. We therefore applied supervised discriminant models and machine learning techniques in subsequent sections to more accurately identify the key stage-dependent chemical markers. The HCA dendrogram further supported the PCA results (Fig. 2D), showed that samples from different storage years were grouped into several major branches, which indicates significant intergroup differences in volatile composition. Notably, the clustering pattern did not strictly follow a linear chronological sequence, but instead revealed a stage-dependent distribution trend. These results suggest that the aroma evolution of QLT during aging is characterized by stage-specific restructuring rather than by a simple monotonic progression. Similar stage-dependent differentiation in volatile profiles has also been reported for stored dark tea and Pu-erh tea products (Shen et al., 2023). PLS-DA has been widely used in tea aroma and metabolomics studies to improve group discrimination and identify differential variables associated with storage, fermentation, or geographic origin (Ho et al., 2015). To further improve discrimination among groups with different storage years, PLS-DA was subsequently performed (Fig. 2E), and the robustness of the model was validated using 1000 permutation tests (Fig. S1). The PLS-DA score plot showed clear separation among QLT samples, indicating marked differences in their volatile profiles. Taken together, the results of PCA, HCA, and PLS-DA consistently demonstrated significant differences in volatile composition among QLT samples stored for different years. In summary, the comprehensive volatile profiling and multivariate statistical analyses demonstrate that the QLT samples are explicitly categorized into three distinct aging stages: the early stage (Q2, Q4), the middle stage (Q6, Q9, Q12, Q14), and the late stage (Q18, Q20, Q25). It should be noted that this specific temporal classification is strictly limited to the samples investigated in this study. These findings provide a reliable multivariate statistical basis for the subsequent screening of differential volatile compounds and the identification of key aroma-active markers associated with QLT aging.

### 3.3 Identification of key aroma-active compounds and their associations with sensory attributes

**Fig. 3:**
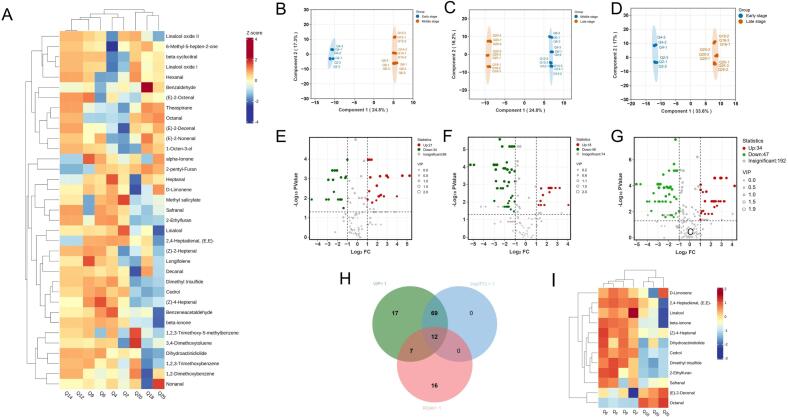
Identification of key differential volatile compounds across different aging stages of QLT. (A) Heatmap showing the distribution patterns of key active-aroma compounds in QLT samples across different aging stages. (B) OPLS DA score plot showing the separation between early and middle aging stage samples. (C) OPLS DA score plot showing the separation between middle and late aging stage samples. (D) OPLS-DA score plot showing the separation between early and late aging stage samples. (E) Volcano plot of volatile compounds differing between early and middle aging stages. (F) Volcano plot of volatile compounds differing between middle and late aging stages. (G) Volcano plot of volatile compounds differing between early and late aging stages. (H) Venn diagram of differential volatile compounds screened using VIP > 1, |log2FC| > 1, and ROAV >1. (I) Heatmap of representative key differential volatile compounds distinguishing early and late aging stages of QLT.

The aroma characteristics of tea are determined by the combined effects of the concentrations and relative proportions of volatile compounds. The contribution of each individual aroma compound to the overall aroma profile of tea largely depends on its ROAV (Li et al., 2026). To identify the compounds that contributed most to the aroma characteristics of QLT, the relative odor activity values of volatile compounds were further calculated (Table 1), resulting in the identification of 35 key volatile aroma compounds. Among them, several lipid oxidation-related compounds, including hexanal, (*E*)-2-decenal, (*E*)-2-nonenal, (*E*)-2-octenal, 1-octen-3-ol, and 2-pentylfuran, exhibited relatively high contributions in some early and middle stage storage samples, whereas their importance generally declined in late-stage samples. These compounds are commonly associated with fresh, green, fatty, or mushroom-like aromas, suggesting that they mainly contribute to the relatively fresh and less aged aroma profile of QLT (Ho et al., 2015). Notably, octanal exhibited a completely distinct, stage-dependent accumulation pattern. It was not detected in the early-stage samples, but its content increased drastically to approximately 63–65 μg/kg in the late-stage samples, suggesting its vital contribution to the specific aged aroma profile of QLT during long-term storage. In contrast, several compounds associated with mature or aged aroma characteristics, including methoxybenzene derivatives, linalool oxides, *β*-ionone, dihydroactinidiolide, and safranal, became more prominent in some middle- and late-stage storage samples, suggesting an increased contribution of woody, floral, sweet, and aged aroma notes after prolonged storage. Carotenoid-derived compounds, such as *β*-ionone and dihydroactinidiolide, are well-recognized contributors to the floral, sweet, and mature aroma of tea, whereas methoxybenzene-related compounds have been consistently associated with aged aroma formation in dark tea (Li et al., 2024; Wen et al., 2023). As shown in the heatmap (Fig. 3A), aroma-active compounds exhibited a clear storage-dependent variation pattern rather than a uniform monotonic trend, suggesting that the sensory differentiation of QLT during aging was driven by the dynamic redistribution of multiple odorants. The clustering results were consistent with those obtained from the HCA dendrogram. In addition, the heatmap revealed that samples from different storage stages did not possess identical sets of aroma-active compounds. For instance, methoxybenzene-related compounds, including 1,2-dimethoxybenzene, 1,2,3-trimethoxybenzene, 1,2,3-trimethoxy-5-methylbenzene, and 3,4-dimethoxytoluene, were particularly prominent in some middle- and late-stage storage samples, whereas in other aged samples, cedrol, ionone-related compounds, or dihydroactinidiolide made relatively greater contributions. These findings indicate that the aroma evolution of QLT during storage is structurally complex and stage-dependent, rather than resulting from a simple linear accumulation of a single odorant. Overall, the ROAV analysis suggested that the aroma differences among QLT samples stored for different years were mainly associated with changes in the relative importance of several key odor groups, including lipid-derived aldehydes and furans, oxygenated terpenoids, carotenoid-derived compounds, and methoxybenzene derivatives.

**Table 1:** Key aroma-active compounds with ROAV ≥1 in QLT.

**No.**	**Key aroma-active** **compounds**	**CAS**^a^	**OT** **(**μ**g/kg)**^**b**^	**Q25**	**Q20**	**Q18**	**Q14**	**Q12**	**Q9**	**Q6**	**Q4**	**Q2**	**Odor description**^**c**^
1	2-Ethylfuran	3208-16-0	2.3	n.d.	n.d.	n.d.	9.86	10.18	13.23	11.94	11.07	3.36	Burnt, sweet, beany, earthy, malty
2	Hexanal	66–25-1	4.5	15.80	17.67	17.64	17.70	18.50	15.00	12.80	10.92	10.80	Grassy, green, fresh, fatty
3	(*Z*)-4-Heptenal	6728-31-0	0.8	6.67	8.09	12.04	13.50	15.77	22.89	30.10	18.26	15.79	Fatty, oily, green, creamy
4	Heptanal	111–71-7	2.8	7.01	8.47	8.72	10.47	11.57	12.22	12.29	12.20	12.29	Heavy, plant green odor, apricot-like, and nutty aroma
5	(*Z*)-2-Heptenal	57,266–86-1	12.21	0.47	0.92	0.98	1.18	1.90	1.93	1.94	1.27	0.69	Green
6	Benzaldehyde	100–52-7	14	1.05	1.37	1.95	1.19	1.60	1.59	1.17	1.27	1.68	Almond-like, fruity, cherry-like, powdery, nutty
7	Dimethyl trisulfide	3658-80-8	2.68	n.d.	n.d.	n.d.	0.74	0.86	1.01	0.90	1.03	0.57	Bitter
8	1-Octen-3-ol	3391-86-4	1	6.91	7.89	8.48	8.67	13.04	11.54	11.72	8.56	8.14	Earthy, green, oily, vegetative-like, fungal
9	6-Methyl-5-hepten-2-one	110–93-0	50	1.17	1.53	1.46	1.35	1.90	1.23	1.14	1.01	0.94	Fruity, apple-like, musty
10	Furan, 2-pentyl-	3777-69-3	6	9.04	13.05	13.92	14.75	17.34	13.50	13.76	10.45	10.25	Fruity, green, earthy beany with vegetable like
11	Octanal	124–13-0	0.7	63.53	65.47	63.02	69.65	81.38	n.d.	n.d.	n.d.	n.d.	Pungent, fruity and floral odor
12	2,4-Heptadienal, (*E,E*)-	4313-03-5	15.4	n.d.	0.69	0.97	1.14	1.99	2.89	3.06	3.06	3.37	Fatty, green, oily, cinnamon-like
13	d-Limonene	5989–27-5	10	6.69	6.07	4.11	8.03	5.33	8.01	6.49	8.34	4.98	Fruity, lemon-like
14	Benzeneacetaldehyde	122–78-1	4	n.d.	0.68	0.59	n.d.	1.17	1.31	2.40	2.87	0.87	Sweet and fragrant honey
15	(*E*)-2-Octenal	2548-87-0	3	5.73	7.16	7.41	12.54	14.34	14.22	13.21	9.17	8.70	Fresh, cucumber-like, fatty, green, herbal, leafy
16	Linalool oxide I	5989-33-3	6	4.52	15.38	3.58	6.42	13.73	9.53	9.58	6.76	8.26	Sweet, floral, creamy
17	Linalool oxide II	34,995–77-2	6	n.d.	13.78	2.09	4.19	13.63	9.69	7.18	n.d.	12.85	Sweet, floral, creamy
18	Linalool	78–70-6	6	n.d.	4.20	4.78	5.62	8.92	9.44	9.23	9.75	23.74	Floral, sweet, grape-like, woody
19	Nonanal	124–19-6	1	173.36	155.27	150.27	160.53	136.12	140.23	120.67	136.82	114.23	Floral, fatty, green, lemon-like
20	1,2-Dimethoxybenzene	91–16-7	3.17	3.49	7.14	2.24	4.80	7.38	5.56	5.16	4.82	5.16	Stale
21	(*E*)-2-Nonenal	18,829–56-6	0.4	20.92	28.40	31.63	44.29	40.90	30.88	40.68	18.60	21.62	Green and tallow-like odor
22	Methyl salicylate	119–36-8	40	0.07	0.06	0.07	0.08	0.23	0.26	0.39	0.38	2.20	Green
23	Safranal	116–26-7	3	4.30	6.23	6.67	12.05	17.76	16.63	17.17	12.96	12.15	Woody, spicy, medicinal, powdery, and herbal
24	Decanal	112–31-2	0.1	202.32	209.37	234.29	267.33	265.63	233.21	194.14	196.49	173.59	Aldehyde-like, candle-like, fatty and citrus-like aroma
25	*β*-cyclocitral	432–25-7	3	4.24	9.57	4.08	6.63	12.70	10.82	11.46	6.99	6.84	Herbal, rose-like, fruity
26	3,4-Dimethoxytoluene	494–99-5	2.71	2.06	12.01	1.69	5.64	5.19	4.60	6.07	4.98	1.57	Stale
27	(*E*)-2-Decenal	3913-81-3	0.4	39.46	38.92	19.45	36.00	79.14	20.47	18.91	7.55	n.d.	Green, fatty
28	Theaspirane	36,431–72-8	0.2	12.36	11.87	15.09	21.25	20.51	11.63	9.78	8.27	6.83	Honey
29	1,2,3-Trimethoxybenzene	634–36-6	0.75	8.70	120.94	6.30	36.20	56.27	56.68	32.97	38.08	20.66	Stale
30	1,2,3-Trimethoxy-5-methylbenzene	6443-69-2	4.45	0.22	6.46	0.61	7.46	2.19	2.65	2.41	2.66	0.47	Stale
31	Longifolene	475–20-7	2	1.75	2.66	2.93	4.79	4.13	4.94	4.27	3.46	3.11	Woody
32	*α*-Ionone	127–41-3	0.4	62.36	82.07	39.01	99.21	126.49	121.30	103.17	71.97	71.43	Floral, violet-like, powdery, berry-like
33	*β*-ionone	79–77-6	0.007	n.d.	1094.34	770.65	2642.62	5001.86	5167.52	6520.36	4878.96	3656.14	Floral, woody, sweet, fruity, berry
34	Dihydroactinidiolide	17,092–92-1	3.8	1.30	5.92	1.26	5.00	14.44	12.45	14.93	11.02	8.42	Woody
35	Cedrol	77–53-2	0.5	14.83	28.56	61.84	66.56	97.00	125.54	147.20	84.92	225.04	Mild cedar wood-like aroma

a CAS, the published chemical abstracts service (CAS) of compounds in NIST 20 library. ^b^ OT(odor threshold) (μg/kg): Odor thresholds in water. These values were sourced from published literature or relevant websites. ^c^ The odor description referred to the database (The Good Scents Company Information System, http://www.thegoodscentscompany.com/).

**Fig. 4:**
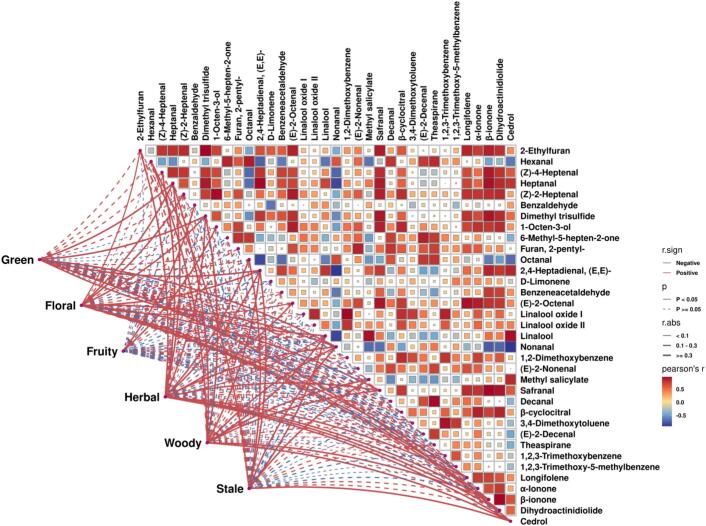
Associations between QDA aroma attributes and key aroma-active compounds of QLT.

To further elucidate the sensory correlations of key odorants, correlation analysis was performed between the QDA sensory attributes and the selected key volatile compounds (Fig. 4). Correlation-based flavoromics has been increasingly applied in tea research to associate aroma-active compounds with sensory descriptors and to identify the compounds responsible for specific aroma attributes (Xue et al., 2026). The sensory–volatile network revealed extensive associations between aroma descriptors and key odorants, suggesting that the characteristic aroma of QLT is determined by the synergistic interactions of multiple volatile compounds rather than by individual components. Among the six sensory attributes, woody and stale notes exhibited the most frequent and strongest positive correlations with various volatile compounds. In particular, these attributes were closely associated with methoxybenzene derivatives, cedrol, longifolene, and several carotenoid-derived compounds, including *β*-ionone and dihydroactinidiolide. Previous studies on dark tea and other stored tea products have likewise shown that cedrol, *β*-ionone, and methoxybenzene-related volatiles are closely associated with the development of woody and stale aroma characteristics (Shen et al., 2024). This observation was also consistent with the sensory results of the present study, in which older samples exhibited more pronounced woody and stale characteristics. In contrast, green and fruity attributes were more strongly associated with a different group of compounds, including several aldehydes and furans, such as hexanal, heptanal, nonanal, 2-ethylfuran, and 2-pentylfuran. These compounds are generally associated with fresher, greener, and more volatile top-note characteristics and have been widely reported as lipid oxidation products during tea aroma formation (Shen et al., 2024). Floral and herbal attributes showed relatively dispersed association patterns, with notable correlations involving compounds such as linalool, linalool oxide I, linalool oxide II, and methyl salicylate. Terpenoid alcohols and their oxidation products are widely recognized as important contributors to floral and herbal notes in tea (Ho et al., 2015).

Notably, 1,2-dimethoxybenzene, 3,4-dimethoxytoluene, 1,2,3-trimethoxybenzene, and 1,2,3-trimethoxy-5-methylbenzene exhibited strong positive correlations with one another. This pattern suggests that these compounds tend to co-accumulate during storage, further supporting their potential role in shaping the characteristic aged aroma of QLT. Previous studies have likewise highlighted the importance of methoxybenzene formation in dark tea, where microorganisms and hygrothermal effects have been shown to strongly influence methoxybenzene-related metabolic pathways (Li et al., 2024). In addition, *β*-ionone, *α*-ionone, dihydroactinidiolide, safranal, and theaspirane may collectively contribute to the floral, sweet, and aged aroma background of aged samples. Carotenoids are important aroma precursors in tea, and their degradation products, including ionones and dihydroactinidiolide, are widely recognized as key contributors to floral and sweet aroma notes (Kirkwood et al., 2025). In addition, several aldehydes and furans, including hexanal, octanal, decanal, and 2-pentylfuran, exhibited coordinated variation patterns, suggesting that the green and fresh aroma dimension was also regulated by a group of co-varying lipid-derived compounds.

Taken together, these results indicate that the aroma differentiation of Qianliang tea across different storage years is jointly driven by sensory-volatile interactions and coordinated changes among volatile modules. In particular, methoxybenzene-related compounds, carotenoid-derived volatiles and selected oxidized terpenoid products appear to play important roles in this process.

### 3.4 Screening of key differential volatile compounds across different aging stages

Based on the stage-dependent clustering patterns, the volatile remodeling of QLT inherently represents a continuous and fluctuating metabolic process. To systematically map these transient metabolic fluctuations, orthogonal partial least squares discriminant analysis models were initially constructed to evaluate the sequential transitions involving the intermediate aging stage. The score plots demonstrated clear spatial separation between the early and middle stages (Fig. 3B) as well as between the middle and late stages (Fig. 3C). Both discriminant models exhibited excellent predictive capability and robustness without overfitting, supported by high permutation test metrics with R2Y and Q2 values exceeding 0.99 (Fig. S2). The corresponding volcano plots visually detailed the dynamic shifts (Fig. 3E-F). Specifically, 55 differential volatile compounds were identified in the transition from the early to the middle stage, and 66 differential compounds were revealed from the middle to the late stage. Cross referencing these two consecutive sets of metabolites successfully identified the continuous or fluctuating trajectories of several common aroma-active compounds. For instance, classic floral volatiles such as linalool exhibited a continuous decline across both transitions, whereas compounds like 2-ethyl-5-methylfuran, methyl benzoate, and ethyl octanoate displayed distinct fluctuating or parabolic accumulation patterns specific to the middle transitional phase.

While the sequential comparisons successfully captured the intermediate metabolic fluctuations, identifying the primary chemical markers driving the overall long-term aging requires a focused contrast between the extreme stages. Therefore, the samples were further classified to directly compare the early and late aging stages, whereas the middle stage was excluded from this specific binary comparison to highlight the major long-term aroma transition. This focused strategy has been widely adopted in tea and food metabolomics studies to improve the interpretability of group discrimination and differential feature screening (Chen et al., 2024). As shown in Fig. 3D, significant differences in volatile compounds were observed between QLT samples from the early and late aging stages. Moreover, the permutation test yielded high R2Y (0.998) and Q2 (0.993) values, while the permuted models performed markedly worse than the original model, confirming that the OPLS-DA model was robust and not overfitted (Fig. S2). These results indicate that the early and late aging stages of QLT can be effectively discriminated on the basis of their volatile profiles, thereby providing a reliable foundation for the subsequent screening of key differential volatile compounds.

To pinpoint the specific volatile compounds most closely associated with the aroma divergence between the aging stages, a joint screening strategy was applied to the dataset. The selection criteria strictly required compounds to exhibit a variable importance in projection value greater than 1, a statistical *p*-value of less than 0.05, and an absolute log2 fold change greater than 1. Following this rigorous statistical filtering, the potential sensory relevance of the remaining differential compounds was further evaluated using relative odor activity values. The fundamental rationale for integrating these strict statistical thresholds with odor activity metrics is to effectively eliminate uninformative background noise and minor random fluctuations. By applying this comprehensive strategy, the massive dataset was successfully narrowed down to highlight the key chemical markers that are both statistically significant and potentially sensorially relevant to the long-term aging process (Xue et al., 2022). Based on these criteria, 12 compounds were identified as key differential volatile compounds (Fig. 3G-H). These compounds clearly discriminated earlier samples (Q2, Q4, Q6, and Q9) from later samples (Q18, Q20, and Q25) (Fig. 3I). Among them, *d*-limonene, linalool, *β*-ionone, dihydroactinidiolide, safranal, cedrol, dimethyl trisulfide, 2-ethylfuran, (*Z*)-4-heptenal, and (*E*,*E*)-2,4-heptadienal exhibited relatively higher abundance or odor contributions in the early aging stage, suggesting that they may serve as potential markers of earlier aroma characteristics. These compounds were mainly associated with floral, citrus-like, sweet, green, and fresh odor dimensions, indicating that early-stage QLT retained a relatively active and diverse volatile profile (Ho et al., 2015). Terpenoid compounds such as d-limonene and linalool are well-established contributors to floral and citrus-like notes in tea, whereas *β*-ionone, safranal, and dihydroactinidiolide are representative carotenoid-derived odorants that contribute to floral and sweet aroma characteristics (Shen et al., 2023). In contrast, octanal and (*E*)-2-decenal exhibited the opposite pattern, with relatively higher levels in the late aging stage, particularly in Q25, suggesting that these compounds may serve as important markers of late-stage aroma remodeling. Therefore, these compounds may be regarded as functional aged aroma markers.

### 3.5 Machine learning-assisted identification of stage-related aroma markers

Machine learning has increasingly been applied in food flavoromics and tea quality studies for classification, marker discovery, and pattern extraction from high-dimensional volatile datasets, while SHAP analysis is considered a useful tool for interpreting the contribution of individual variables to model output (Ge et al., 2025; Wu et al., 2025). To further validate the stage-discriminative potential of the key odorants, machine learning models were established using the 35 compounds with ROAV>1 as input variables.

**Fig. 5:**
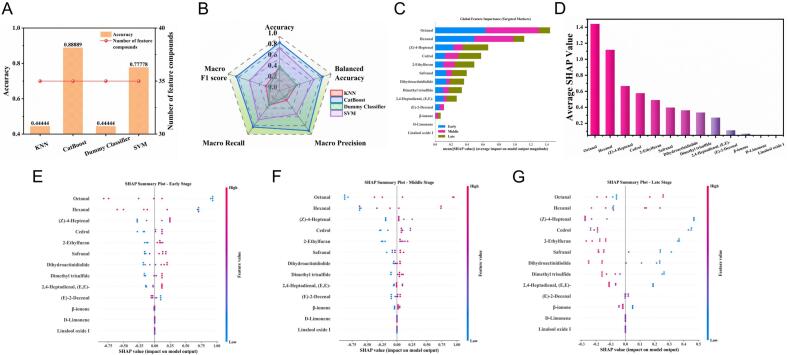
Machine learning-based discrimination of storage stages and SHAP interpretation of targeted aroma markers in QLT. (A) Classification accuracy of different machine learning models (KNN, CatBoost, Dummy Classifier and SVM) based on 35 aroma-active compounds with ROAV>1. (B) Radar plot comparing model performance in terms of accuracy, balanced accuracy, macro precision, macro recall, and macro F1 score. (C) Global SHAP feature importance of the targeted aroma markers, showing the average contribution of each compound to the classification of Early, Middle, and Late storage stages. (D) Average SHAP values of the major targeted markers ranked by overall contribution to model output. (*E*-G) SHAP summary plots showing the stage-specific effects of targeted aroma markers on model prediction for the Early stage (E), Middle stage (F), and Late stage (G), respectively. Each point represents one sample; the x-axis indicates the SHAP value, reflecting the impact of a given compound on model output, and the color represents the relative feature value from low to high.

Among the tested classifiers, CatBoost achieved the best overall performance, with an accuracy of 89%, outperforming KNN and SVM (both 78%) and showing a clear advantage over the dummy classifier (44%) (Fig. 5A). The radar plot further indicated that CatBoost performed better in terms of balanced accuracy, macro precision, macro recall, and macro F1 score, suggesting that the selected aroma-active compounds contained robust stage-related information rather than random classification signals (Fig. 5B). Ensemble learning models have been reported to perform particularly well in complex food volatile datasets because of their ability to capture nonlinear interactions among variables (Feng et al., 2024; Ge et al., 2025).

Machine learning-assisted feature selection strategies have been successfully used to identify key volatile markers in tea and other food systems (Shao et al., 2025). SHAP analysis of the optimal model showed that stage discrimination was mainly driven by a limited number of highly informative compounds rather than by all variables equally (Fig. 5C-D). Octanal and hexanal exhibited the highest mean absolute SHAP values, followed by (*Z*)-4-heptenal, cedrol, 2-ethylfuran, safranal, dihydroactinidiolide, dimethyl trisulfide, (*E,E*)-2,4-heptadienal, and (*E*)-2-decenal (Fig. 5E-G). This result is consistent with the previous ROAV-based screening and multivariate statistical analyses, suggesting that the compounds highlighted by statistical and sensory approaches were also the most informative variables for machine learning-based stage discrimination.

Stage-specific SHAP plots further revealed that (*Z*)-4-heptenal, cedrol, 2-ethylfuran, safranal, and dihydroactinidiolide contributed more positively to the Early stage, whereas octanal and hexanal showed the strongest positive contributions to the Late stage. By contrast, the Middle stage exhibited a more transitional SHAP distribution pattern, indicating that it represented an intermediate aroma remodeling phase. These findings suggest that machine learning not only improved storage-stage discrimination, but also provided additional support for identifying stage-related aroma markers of QLT during aging. Furthermore, by linking these machine learning outputs back to the sensory correlation network established earlier, a direct mapping between the key chemical markers and specific aroma profiles is successfully achieved. This integration effectively bridges the critical gap between statistical feature selection and actual sensory relevance.

### 3.6 Proposed aroma evolution patterns of key volatile compounds during aging

By integrating the results of ROAV evaluation, sensory-volatile correlation, differential screening, and machine learning-based interpretation, the aroma evolution of QLT during aging could be summarized as coordinated remodeling of several volatile groups, rather than a single confirmed biochemical pathway. In tea aroma research, volatile compounds are commonly discussed in relation to major precursor systems such as lipids, terpenoids, carotenoids, glycosides, and aromatic precursors, but their actual behavior during storage is often dynamic and overlapping (Ho et al., 2015; Li et al., 2025; Zheng et al., 2026).

A first pattern involved lipid oxidation-related volatiles, including compounds such as hexanal, heptanal, octanal, nonanal, (*Z*)-4-heptenal, (*E*)-2-nonenal, (*E*)-2-decenal, (*E,E*)2,4-heptadienal, 1-octen-3-ol, and 2-pentylfuran, which are widely associated with green, fresh, fatty, or mushroom-like odor impressions in tea (Ho et al., 2015). In the present study, several of these compounds showed stronger odor contribution or discriminative relevance in early-stage samples, whereas octanal and (E)-2-decenal remained relatively more prominent in part of the late-stage samples. This suggests that lipid-derived odorants in QLT did not undergo a uniform decline during aging. In contrast,，some early stage fresh and green volatiles weakened, while a limited number of aldehydes were selectively retained or became more informative for late stage classification (Pu et al., 2025).

A second pattern involved terpenoid and oxygenated terpenoid-related volatiles. Compounds such as *d*-limonene and linalool were more characteristic of the early stage, suggesting that they contributed to a relatively vivid floral and citrus-like aroma background at the beginning of storage (Ho et al., 2015). By contrast, oxygenated terpenoids such as linalool oxide I and linalool oxide II showed more stage-dependent or sample-dependent variation, implying that the aging of QLT may involve not only the decline of fresh terpenes but also the restructuring of oxygenated floral/herbal notes. This interpretation is generally consistent with recent tea aroma studies showing that terpenoid balance, rather than the absolute abundance of one terpene alone (Ming et al., 2025).

A third pattern was associated with carotenoid-derived odorants, including β-ionone, α-ionone, *β*-cyclocitral, safranal, theaspirane, dihydroactinidiolide, and geranylacetone, which are widely regarded as contributors to floral, fruity and sweet aroma notes in tea (Liu et al., 2025). In the present study, several of these compounds, particularly *β*-ionone, dihydroactinidiolide, safranal, and theaspirane, showed clear stage-related contributions in the ROAV profile and machine learning interpretation, while also exhibiting close association with woody, stale, and partially floral sensory attributes. However, their behavior was not strictly monotonic across storage years, suggesting that carotenoid-related aroma evolution in QLT is better interpreted as a dynamic contribution to aged aroma rather than a simple cumulative trend (Kawakami, 2002).

Among the different volatile groups, methoxybenzene-related compounds appeared most consistently linked to the sensory direction of aged aroma. Compounds such as 1,2-dimethoxybenzene, 3,4-dimethoxytoluene, 1,2,3-trimethoxybenzene, and 1,2,3-trimethoxy-5-methylbenzene formed a strongly correlated module in the interaction network and were closely associated with woody and stale attributes. Recent studies on Pu-erh tea and dark tea have repeatedly emphasized the importance of methoxybenzene derivatives in aged aroma expression, especially in relation to woody and stale-like sensory notes (Liu et al., 2025; Ma et al., 2025). Although these compounds are not consistently identified as key differential metabolites among dark tea samples of different ages, they may better indicate the trajectory of aroma evolution (Chen et al., 2024).

Furthermore, the unique compression and traditional multi-layer packaging techniques of QLT play an indispensable role in shaping its distinct aroma evolution, particularly driving the late-stage accumulation of methoxybenzene derivatives. QLT is tightly pressed into a dense column and sequentially wrapped in layers of polygonum leaves, brown leaves, and a woven bamboo basket. This specialized packaging creates a distinct, semi-closed micro-environment that finely regulates internal water activity and limits oxygen permeation over years of storage. According to comprehensive microbiome studies on Anhua dark teas, this specific environment with low water activity and high polyphenol stress selectively favors the colonization and dominance of xerophilic and polyphenol-tolerant fungi, particularly Eurotium and Aspergillus species (Li et al., 2017). Crucially, the complex enzymatic systems secreted by these specific microorganisms are the primary drivers of late-stage flavor refinement. Microbiological and metabolic mechanisms in post-fermented teas indicate that microbial esterases and tannases first degrade complex catechins to release phenolic precursors such as gallic acid. Subsequently, microbial metabolic pathways actively catalyze the methylation of these precursors (Liu et al., 2024). Through this specific biotransformation pathway, phenolic precursors are continuously converted into volatile methoxybenzene derivatives, such as 1,2,3-trimethoxybenzene and 1,2-dimethoxybenzene. These compounds have been universally confirmed as the quintessential markers for the aged aroma of dark teas (Chen et al., 2024). Consequently, the sustained microbial methylation within this uniquely packaged environment provides a definitive chemical mechanism for the formation of the characteristic woody, stale, and aged aroma unique to long-term aged QLT.

Taken together, the aging-related aroma evolution of QLT could be summarized as a coordinated process involving the attenuation or redistribution of part of the lipid-derived fresh and green odorants, the restructuring of terpenoid-related floral and herbal notes, the dynamic contribution of carotenoid-derived mature odorants, and the increasing sensory importance of methoxybenzene-related aged-aroma volatiles. This interpretation helps explain why the aroma profile gradually shifts from a relatively fresh and active expression in the early stage toward a more integrated woody, stale, and herbal profile in the late stage.

## 4 Conclusion

Due to its unique processing and packaging techniques, QLT exhibits complex and dynamic aroma changes during long-term storage. The aroma evolution characteristics and key chemical basis of QLT under different storage years were systematically revealed using sensory evaluation, GC × GC-QTOFMS volatile metabolomics, multivariate statistical analysis, and machine learning. The results demonstrated that QLT gradually shifted from a green and fresh sensory profile in the early stage to a woody, stale, and herbal profile in the late stage. This transition was mainly associated with dynamic changes in lipid oxidation-related volatiles, carotenoid-derived odorants, oxygenated terpenoids, and methoxybenzene derivatives. Differential screening and SHAP-based interpretation convergently identified octanal, hexanal, (*Z*)-4-heptenal, cedrol, safranal, and dihydroactinidiolide as major contributors to storage-stage discrimination, while linalool, *β*-ionone, *d*-limonene, dimethyl trisulfide, 2-ethylfuran, (*E,E*)-2,4-heptadienal, and (*E*)-2-decenal further supported stage differentiation. Overall, the present work does not directly verify biochemical pathways, but it supports a stage-dependent aroma remodeling framework for QLT aging and provides a useful basis for storage-stage identification, aroma marker screening, and quality evaluation of aged QLT.

## CRediT authorship contribution statement

**Mengzhen Xia:** Writing – original draft, Visualization, Investigation, Formal analysis, Data curation, Conceptualization. **Zhichao Lin:** Writing – original draft, Validation, Investigation, Formal analysis, Data curation. **Guohe Chen:** Validation, Methodology, Investigation, Conceptualization. **Jiazhen San:** Validation, Methodology, Investigation, Formal analysis. **Xiaoxiao Fan:** Validation, Methodology, Data curation, Conceptualization. **Lianqing Wang:** Writing – review & editing, Validation, Methodology, Investigation. **Jianan Huang:** Supervision, Project administration, Funding acquisition, Conceptualization. **Zhonghua Liu:** Supervision, Resources, Project administration, Funding acquisition, Conceptualization. **Chao Wang:** Writing – review & editing, Project administration, Methodology, Funding acquisition, Formal analysis, Data curation.

## Ethical statement

The authors ensure that the work described has been carried out in accordance with The Code of Ethics of the World Medical Association (Declaration of Helsinki) for experiments involving humans. The ethical approval of sensory evaluation is not required by national laws. No human ethics committee or formal documentation process is available for sensory evaluation. The authors confirm that the appropriate protocols for protecting the rights and privacy of all participants were utilized during the execution of the research, including no coercion to participate, full disclosure of study requirements and risks, verbal consent of participants, no release of participant data without their knowledge, and the ability to withdraw from the study at any time.

## Declaration of competing interest

The authors declare that they have no known competing financial interests or personal relationships that could have appeared to influence the work reported in this paper.

## Data Availability

Data will be made available on request.
