# Supplementary material for: Aroma evolution of Anhua Qianliang tea across different storage years: Machine learning-assisted discrimination of storage stages and screening of marker volatile compounds
Source: Food Chem X. 2026 Jun 29;37:104151. doi: 10.1016/j.fochx.2026.104151 (PMC13330534; doi:10.1016/j.fochx.2026.104151)
Supplement: Supplementary file 1 — Supplementary material 1 [file mmc1.docx]

**Supplemental Figure Captions：**

**Figure S1.** Cross-validation and permutation test of the PLS-DA model for QLT samples. (A) Cross-validation plot of the PLS-DA model based on accuracy, R², and Q² values with different numbers of components. (B) Permutation test of the PLS-DA model (1000 permutations), showing that the observed model significantly outperformed the permuted models (p<0.001).

**Figure S2.** Permutation validation of the orthogonal partial least squares discriminant analysis models for QLT samples across different storage stages. (A) Permutation test results for the comparison between early and middle aging stages. (B) Permutation test results for the comparison between middle and late aging stages. (C) Permutation test results for the comparison between early and late aging stages. All models exhibited high goodness of fit and robust predictive performance with R2Y and Q2 values exceeding 0.99. These permutation results confirmed that the established models were statistically valid and not overfitted.


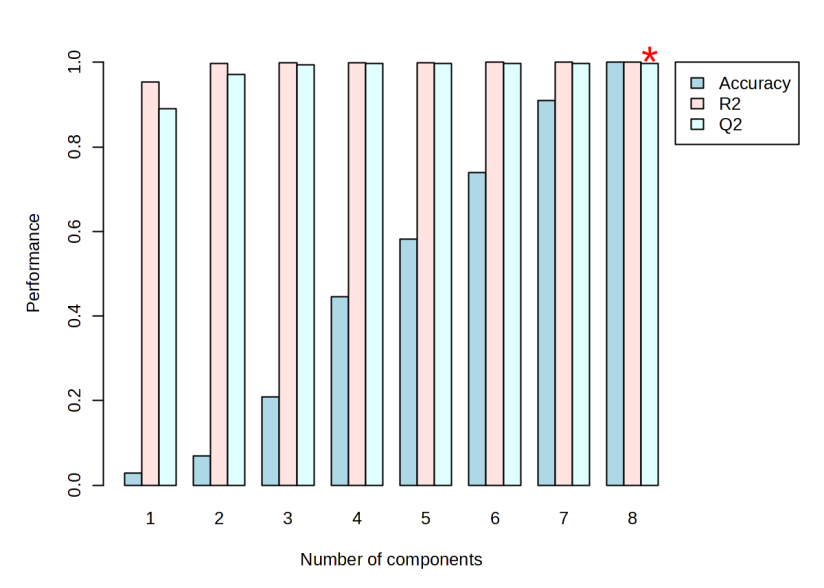


B

A


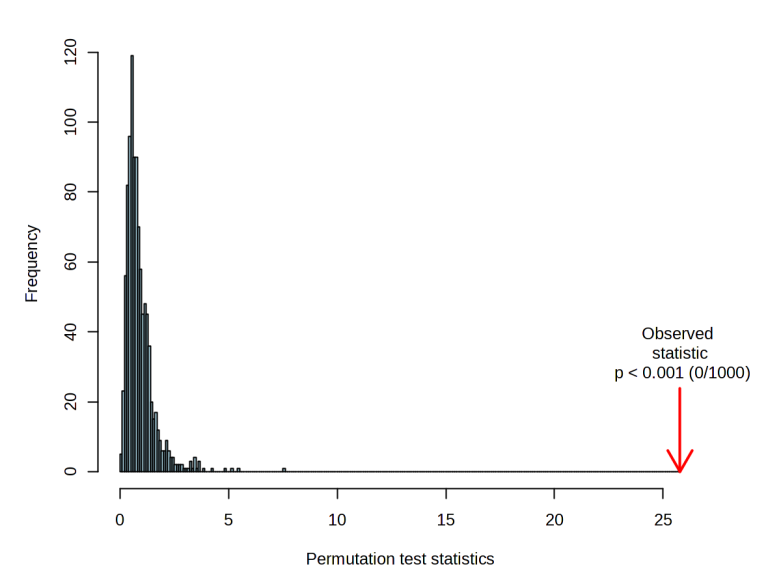


**Figure S1.** Cross-validation and permutation test of the PLS-DA model for QLT samples. (A) Cross-validation plot of the PLS-DA model based on accuracy, R², and Q² values with different numbers of components. (B) Permutation test of the PLS-DA model (1000 permutations), showing that the observed model significantly outperformed the permuted models (p<0.001).


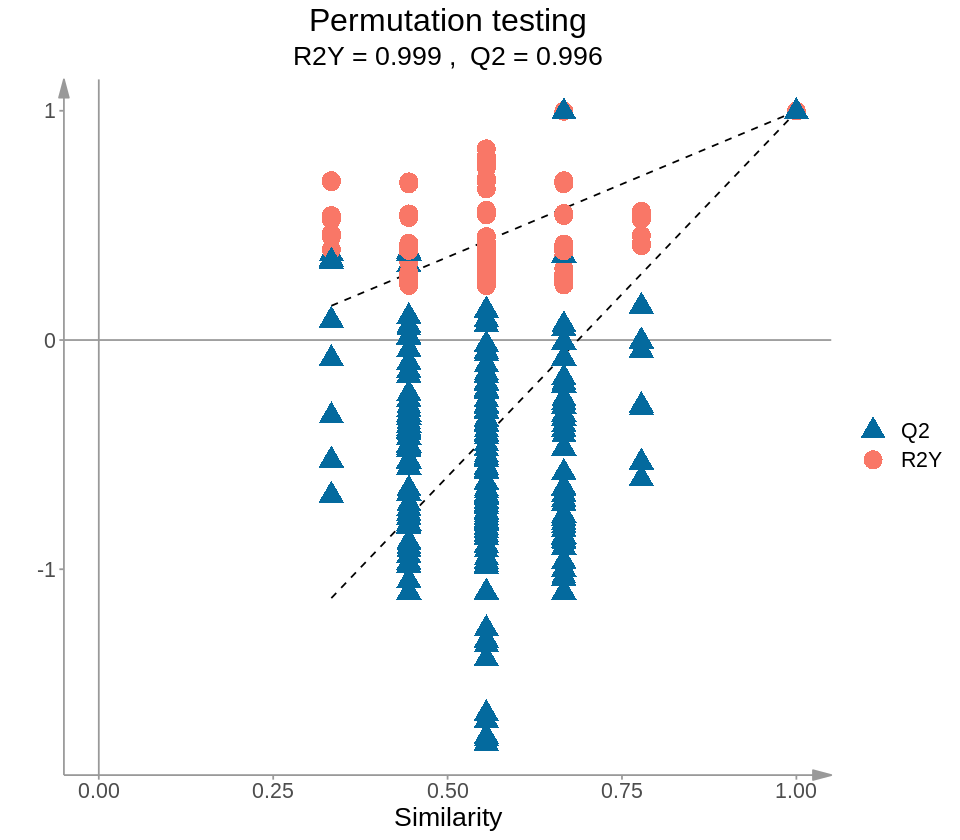


A

B


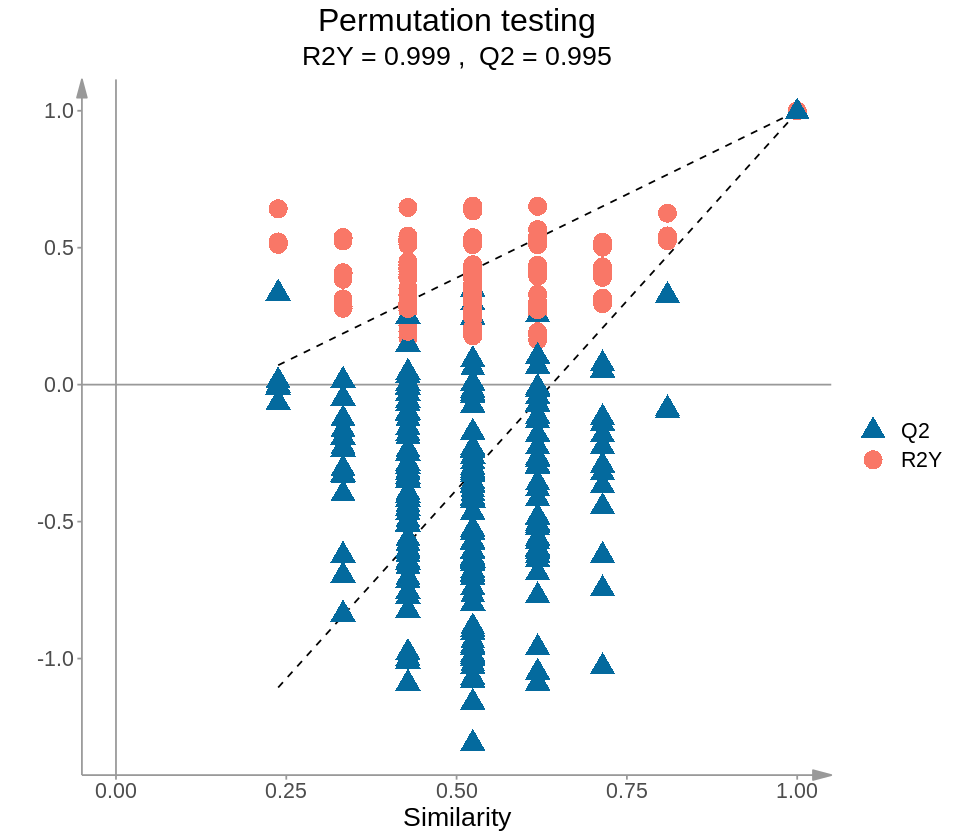


C


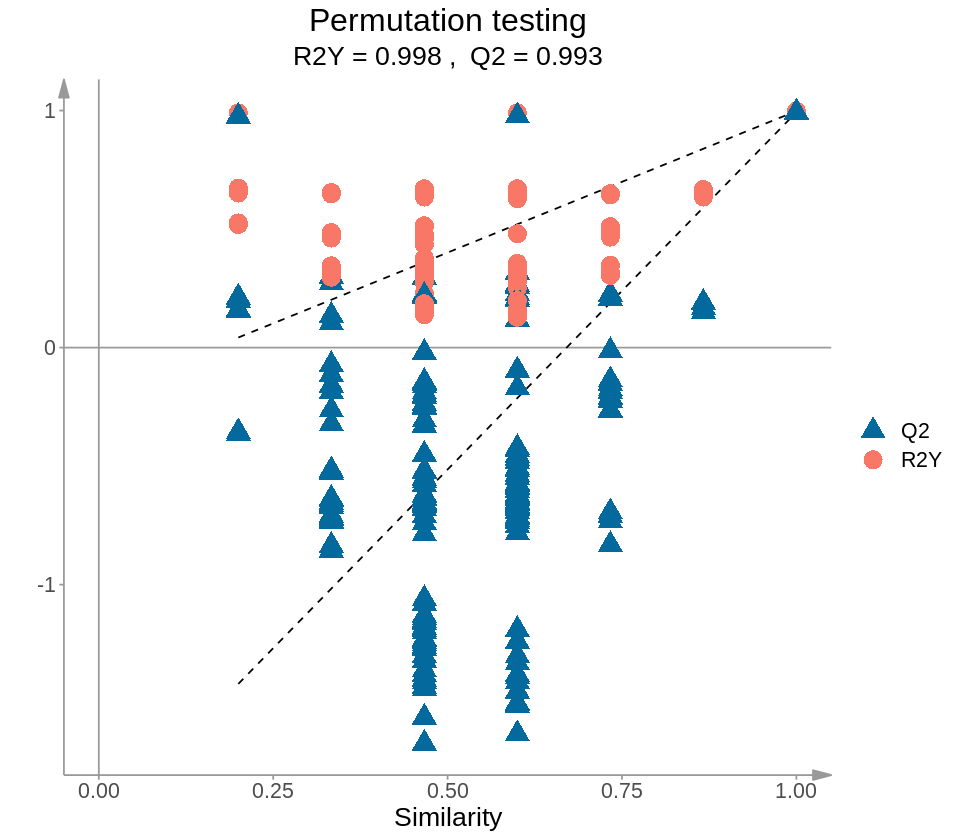


**Figure S2.** Permutation validation of the orthogonal partial least squares discriminant analysis models for QLT samples across different storage stages. (A) Permutation test results for the comparison between early and middle aging stages. (B) Permutation test results for the comparison between middle and late aging stages. (C) Permutation test results for the comparison between early and late aging stages. All models exhibited high goodness of fit and robust predictive performance with R2Y and Q2 values exceeding 0.99. These permutation results confirmed that the established models were statistically valid and not overfitted.
